# SARS-CoV-2 Viral Load in Stool and Nasopharyngeal/Oropharyngeal Samples: Implications for Clinical Progression in Severe COVID-19 Patients

**DOI:** 10.1155/ijm/1501327

**Published:** 2025-10-10

**Authors:** Mariane Vedovatti Monfardini, Brena Ramos Athaydes, Priscila Marinho Abreu, Fernanda Laís Lima Fonseca, Roberta Ferreira Ventura Mendes, Juliana Couto-Vieira, Frederico Firme Figueira, Priscilla Aquino Martins, Regina Keller, Sandra Lucia Ventorin von Zeidler, Liliana Cruz Spano

**Affiliations:** ^1^Department of Pathology, Health Sciences Center, Federal University of Espírito Santo, Vitória, Espírito Santo, Brazil; ^2^Department of Teaching and Research, Hospital Estadual Dr Jayme Santos Neves, Serra, Espírito Santo, Brazil; ^3^Sanitation Laboratory, Department of Environmental Engineering, Federal University of Espírito Santo, Vitória, Espírito Santo, Brazil

**Keywords:** adult, COVID-19, intensive care units (ICUs), reverse transcriptase PCR (RT-qPCR), SARS-CoV-2, viral load

## Abstract

The SARS-CoV-2 RNA load in different specimens has been analyzed to evaluate its correlation with disease outcomes and other factors. However, conflicting results have emerged due to variations in study design. This study examines the impact of viral load in stool and nasopharyngeal/oropharyngeal (NP) specimens on the clinical outcomes of hospitalized patients with severe COVID-19. Forty-six intensive care unit (ICU) patients with COVID-19 were enrolled between September 2020 and March 2021. NP swab and stool samples were collected at admission and at clinical outcome, and viral load was quantified using RT-qPCR. All patients had positive NP RNA at admission, with 41 (89.1%) also testing positive in stool samples. At the time of clinical outcome, 67.4% of NP samples and 45.7% of stool samples remained positive. Patients without gastrointestinal symptoms had a higher stool viral load at admission. Additionally, a greater NP viral load at admission was associated with unfavorable outcomes, whereas patients with diarrhea exhibited a more rapid decline in NP viral load. These preliminary findings suggest that diarrhea is associated with faster NP viral load reduction. Furthermore, viral load clearance was more efficient in stool samples than in respiratory specimens. These results highlight the relationship between viral load and COVID-19 severity.

## 1. Introduction

SARS-CoV-2, the causative agent of severe acute respiratory syndrome coronavirus 2 (COVID-19), has resulted in over 7 million deaths worldwide [1]. The infection spectrum ranges from asymptomatic and mild cases to moderate and severe forms requiring hospitalization, with unfavorable prognosis associated with severe disease [2].

SARS-CoV-2 has been continuously evolving, acquiring beneficial mutations that affect transmissibility, antigenicity, infectivity, and overall viral fitness [3]. Greater genomic diversity has been observed in stool samples compared to nasopharyngeal specimens, suggesting that strain selection and viral replication occur within the gastrointestinal (GI) tract [4].

SARS-CoV-2 gains entry into host cells through the angiotensin-converting enzyme 2 (ACE2) receptor and the transmembrane serine protease 2 (TMPRSS2), both of which are highly expressed on intestinal epithelial cells [5]. Upon binding to these receptors, the virus can invade intestinal cells, triggering a cascade of events that leads to cell death, disruption of tight junctions, and loss of epithelial integrity [6]. These pathological alterations increase intestinal permeability, enabling viral particles or their RNA to be released into the GI tract [6]. This process may elicit a localized immune response—partially mediated by cytokines—that mirrors the systemic inflammation observed in the lungs. Diarrhea is the most frequently reported GI symptom among COVID-19 patients, with an incidence ranging from 10% to 26% [8, 9]. The detection of viral RNA in feces underscores the GI involvement in SARS-CoV-2 infection and holds important implications for epidemiological surveillance, particularly in the context of community-level monitoring efforts [6].

As seen with human immunodeficiency virus (HIV), human papillomavirus (HPV), influenza virus, and hepatitis B and C viruses, where viral load is investigated as a biomarker for disease progression and therapeutic efficacy, SARS-CoV-2 RNA quantification has become widely adopted [10]. Viral RNA load has been measured in nasopharyngeal, stool, and other clinical specimens to assess its potential as a predictor of disease outcome alongside other clinical factors [8, 11–13]. However, cross-study comparisons are challenging due to inconsistencies in measurement units [8, 9, 13, 14]. Moreover, the relationship between stool viral load and disease severity remains controversial [8, 14], highlighting the need for further investigation into viral load dynamics to clarify its role in SARS-CoV-2 pathogenesis.

The evolution of viral load during hospitalization for severe COVID-19 and its association with clinical parameters, hospitalization course, and patient outcomes remain underexplored. Therefore, this study is aimed at analyzing SARS-CoV-2 viral load in stool and nasopharyngeal samples and investigating its clinical implications in hospitalized patients with severe complications.

## 2. Materials and Methods

### 2.1. Study Setting and Clinical Samples

Patients with COVID-19 admitted to the intensive care unit (ICU) of Hospital Dr. Jayme Santos Neves—a designated COVID-19 referral center in Espírito Santo, southeastern Brazil—between September 2020 and March 2021 were enrolled in this study. Confirmation of SARS-CoV-2 infection was based on the detection of viral RNA by reverse transcription polymerase chain reaction (RT-PCR) from nasopharyngeal and/or oropharyngeal (NP) swab samples collected at the time of ICU admission. All samples were obtained prior to study enrollment. Eligibility criteria included symptom onset within the previous 10 days and ICU admission no more than 2 days prior to enrollment. All patients were prospectively followed until a defined clinical outcome was reached.

For each participant, two NP swab samples and two stool specimens were collected. The first set was obtained at ICU admission or shortly thereafter. The NP sample used for COVID-19 confirmation by RT-PCR at the hospital was retained and considered the admission NP sample for our analyses. Given the variability of bowel movements among ICU patients, the admission stool sample was defined as the first specimen collected following study enrollment. The second set of samples was collected at the time of clinical outcome (discharge, death, or transfer) or as close as possible to the end of hospitalization. Stool samples were collected throughout the ICU stay; when it was not feasible to obtain a sample exactly at the outcome timepoint, the one closest to it was used. Inclusion criteria required the collection of all four samples per patient. Patients with prolonged hospitalization (more than 15 days) were excluded if their samples were collected more than 10 days after ICU admission or more than 10 days before the clinical outcome.

A total of 231 adult patients with a confirmed COVID-19 diagnosis (positive PCR test to NP) were recruited. Of these, 69 had two NP swabs and two stool samples collected; however, 23 were excluded due to stool sample collection occurring more than 10 days after admission or outcome. Consequently, 46 patients were included in the final analysis. NP swabs in viral transport medium and stool samples were transported at 4°C to the laboratory at the Federal University of Espírito Santo, processed within 72 h, and stored at −80°C until testing.

Sociodemographic and clinical data, including age, sex, comorbidities, symptoms, hospitalization duration, oxygen support, laboratory findings, and clinical outcomes, were extracted from electronic medical records. This prospective cohort study was conducted in accordance with the principles of the Declaration of Helsinki and was approved by the Ethics and Research Committee of the Health Sciences Center of the Federal University of Espírito Santo (Protocol 4.175.329). Written informed consent was obtained from all participants. All methods and procedures adhered to relevant guidelines and regulations.

### 2.2. RNA Extraction

A 10%–20% suspension of each stool sample was prepared using Tris–calcium buffer (pH 7.2) and processed for RNA extraction. A 140-*μ*L aliquot was subjected to the QIAamp Viral RNA Mini Kit (QIAGEN, Hilden, Germany) following the manufacturer's instructions, and RNA was eluted in a final volume of 60 *μ*L. For NP swab samples, 200 *μ*L of sample was used for RNA extraction using the PureLink Viral RNA/DNA Mini Kit (Invitrogen, Carlsbad, California, United States) according to the manufacturer's instructions and eluted in a final volume of 50 *μ*L.

### 2.3. RT-qPCR and Viral Load

RT-qPCR was performed using specific primers and hydrolysis probes targeting two regions of the SARS-CoV-2 nucleocapsid gene (N1 and N2), along with an internal control (RNase P-RP), as recommended by the Centers for Disease Control and Prevention [15]. Primers and probes were obtained from the 2019-nCoV RUO Kit (Integrated DNA Technologies, IDT; 500 reactions; Catalog #10006713). Reactions were carried out using the TaqPath 1-Step RT-qPCR Master Mix (Thermo Fisher) in a StepOnePlus PCR System (Applied Biosystems). Negative and positive controls (2019-nCoV, IDT) were included in all runs for validation. A result was considered positive if the cycle threshold (Ct) value was ≤ 40 for all three target regions and inconclusive if it was > 40 for any of the regions.

To determine viral load (copy number), the N1 primer/probe set (CDC, IDT) was used for RNA detection. A positive control (IDT) was serially diluted (2.0 × 10^0^–2.0 × 10^5^ copies/*μ*L) to generate a six-point standard curve, which was used in triplicate. The total extracted RNA was quantified in duplicate using the same real-time PCR conditions, and SARS-CoV-2 viral load was calculated per gram of stool or milliliter of NP swab sample.

The mean change in viral load (increase or decrease) per day was calculated for patients with quantifiable viral loads in both admission and outcome samples, using the interval between their respective collection dates. The total days between collections was defined as the difference between the number of days after ICU admission of the outcome sample collection and the admission sample collection. The mean change in viral load per day was calculated using the following formula: mean change in viral load per day (viral load at outcome − viral load at admission)/(total days between sample collections).

### 2.4. Statistical Analysis

Comparisons of dependent continuous variables were performed using the Wilcoxon or Friedman test, while independent variables were analyzed using the Mann–Whitney or Kruskal–Wallis test, followed by post hoc testing when applicable. Proportional comparisons were conducted using Pearson's chi-square or Fisher's exact test, as appropriate. A *p* value < 0.05 was considered statistically significant. All statistical analyses were conducted using the SPSS software package, version 20.0 (IBM, Armonk, New York, United States).

Outliers, defined as values beyond three standard deviations from the mean or exceeding 1.5× the interquartile range, were included in visual representations to capture the full range of variation, as they reflect individual-specific immune responses to the disease. To minimize their influence on statistical analyses, the median was used as a central measure, given its robustness to extreme values. To improve the visualization of data with large ranges, a logarithmic scale (log_10_) was applied to compress extreme values and emphasize proportional differences.

## 3. Results

**Figure 1:**
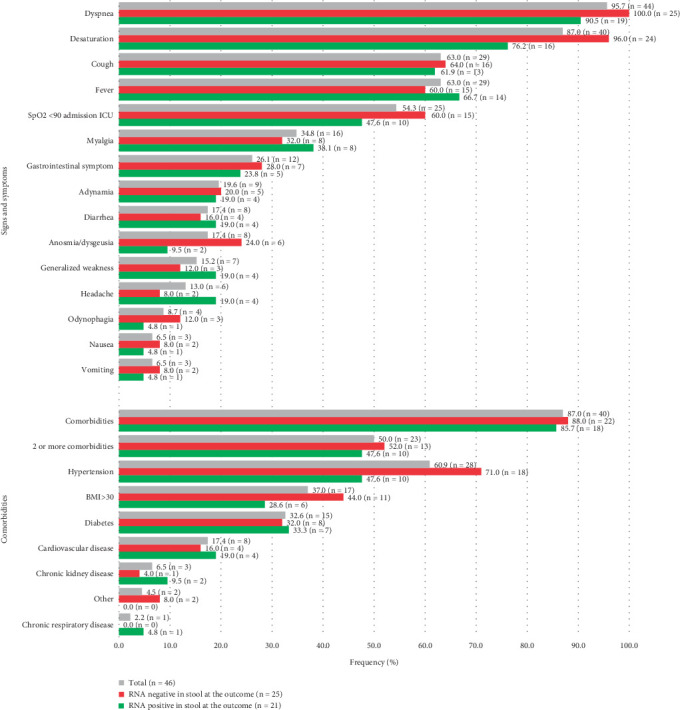
Frequency of signs, symptoms, and comorbidities at ICU admission among 46 patients, categorized by stool RNA detection outcome. This bar chart illustrates the frequency of signs, symptoms, and comorbidities observed at ICU admission in the total cohort of 46 patients. Data are further stratified into two groups based on stool RNA detection outcome: 21 patients with stool RNA detected at outcome (positive group) and 25 patients without stool RNA detected at outcome (negative group). Frequencies are expressed as percentages for each subgroup, followed by the absolute number of participants (*n*) in parentheses. Gastrointestinal symptom: at least one of the symptoms: diarrhea, vomiting, or nausea. Other: one with cancer and one with autoimmune disease.

Over a 7-month period, 46 patients were admitted: 12 in September 2020, five in October 2020, 10 in November 2020, seven in December 2020, nine in January 2021, two in February 2021, and one in March 2021. The median age was 61 years (range: 30–88), with 25 patients (54.3%) being male (Table S1). Six patients (13%) had no comorbidities, while 50% had two or more (Figure 1). The median hospitalization duration during the first 5 months ranged from 12 to 17.5 days, while the remaining three eligible patients were hospitalized for 48, 17, and 41 days.

The median time from symptom onset to ICU admission was 7 days (range: 1–10). The most common clinical symptoms were dyspnea (95.7%), desaturation (87%), fever (63%), and cough (63%) (Figure 1). GI symptoms, including diarrhea, nausea, and/or vomiting, were reported in 12 patients (26.1%), with nausea and vomiting being the least frequent, each occurring in 6.5% of cases (Figure 1). Noninvasive ventilation was the most commonly used oxygen support upon admission. More detailed sociodemographic and clinical data are presented in Table S1.

The median length of hospital stay was 16 days (Table 1). The most frequently used ventilation methods during hospitalization were noninvasive ventilation (58.7%) and orotracheal intubation (OI) (56.5%), with a median duration of 3 and 16 days, respectively (Table 1).

**Figure 2:**
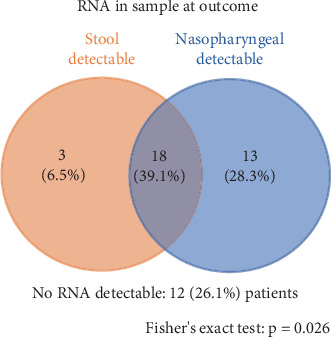
Overlap between patients with stool and nasopharyngeal/oropharyngeal RNA detection. This Venn diagram represents the intersection of patients with RNA detection in stool and nasopharyngeal/oropharyngeal swabs at ICU admission. From the total cohort, 18 patients tested positive in both samples, while 3 tested positive exclusively in stool samples, and 13 tested positive exclusively in nasopharyngeal/oropharyngeal swabs. The remaining 12 patients had negative results for RNA detection in both sample types. Fisher's exact test result for the association between stool RNA and NP RNA detection at outcome, *p* = 0.026.

All patients had a positive NP swab, as per the inclusion criteria, and 41 (89.1%) tested positive for viral RNA in stool samples at admission. At the time of outcome, 21 (45.7%) patients remained positive for stool RNA, while 31 (67.4%) had detectable RNA in NP samples. RNA detection in stool was associated with RNA detection in NP samples at outcome (*p* = 0.026) (Figure 2) (Table S2). Overall, 18 (39.1%) patients tested positive in both specimens at outcome, most of whom (66.7%) had a hospital stay shorter than 15 days (*p* < 0.05).

No significant differences were observed between groups (positive vs. negative stool RNA at outcome) regarding sociodemographic characteristics, comorbidities, symptoms, ventilation support upon admission, or laboratory findings (Table S1). However, patients with prolonged ICU stays and those requiring invasive ventilation were more likely to have negative stool RNA at outcome (*p* < 0.05) (Table 1).

The median number of days between admission and specimen collection was 0 and 4 days for the NP and stool samples at admission and 16 and 14 days at outcome, respectively. The maximum duration of viral RNA shedding was 44 days in NP samples and 48 days in stool samples.

Viral load assays were performed on all 139 positive specimens. Eleven samples were below the quantification limit: one NP and two stool samples at admission and five NP and three stool samples at outcome. The median NP viral load significantly decreased between admission and outcome, by approximately two base-10 logarithms (9.07 × 10^5^ vs. 8.83 × 10^3^ copies/mL, *p* = 0.002, Wilcoxon test). In contrast, the median stool viral load increased by 0.3 base-10 logarithm (1.37 × 10^5^ vs. 2.8 × 10^5^ copies/g, *p* > 0.05).

**Figure 3:**
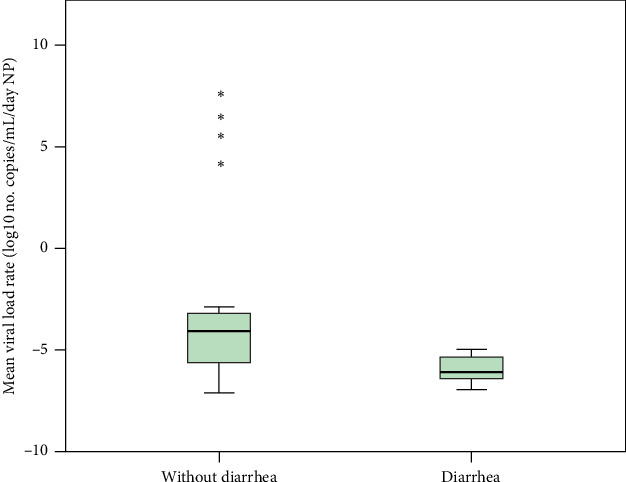
Mean base-10 logarithmic viral load rate per day from nasopharyngeal/oropharyngeal (NP) swab in patients with/without diarrhea. For patients experiencing a decrease in viral load, the base-10 logarithmic value was multiplied by [−1]. *p* = 0.029, Mann–Whitney test. ⁣^∗^Outliers. Viral load measurements are presented on a log_10_ scale to reduce distortion from extreme values and enhance visualization of proportional variations. Outliers are represented to capture the full range of data reflecting individual-specific responses.

The analysis of mean change in viral load was restricted to patients with paired samples of the same specimen type that had quantifiable viral loads. Thus, a viral load increase during hospitalization was observed in 11.5% (3/26), with a median increase of 3.7 × 10^6^ copies/mL/day among the NP samples from 26 patients. Conversely, 23 patients exhibited a median viral load reduction of 1.9 × 10^5^ copies/mL/day. In stool samples, viral load increased in 27.8% (5/18) of patients at outcome (median increase of 5.8 × 10^5^ copies/g/day), whereas 13 patients showed a median reduction of 6.3 × 10^5^ copies/g/day. The mean daily viral load rate was analyzed over time and compared across outcomes, sex, age (> 65 years), and diarrhea symptoms. Patients with diarrhea exhibited a greater reduction in NP viral load than those without diarrhea (*p* = 0.029, 1.27 × 10^6^ vs. 1.72 × 10^4^ copies/mL/day, medians) (Figure 3). No significant differences were observed in stool viral load across these variables.

**Figure 4:**
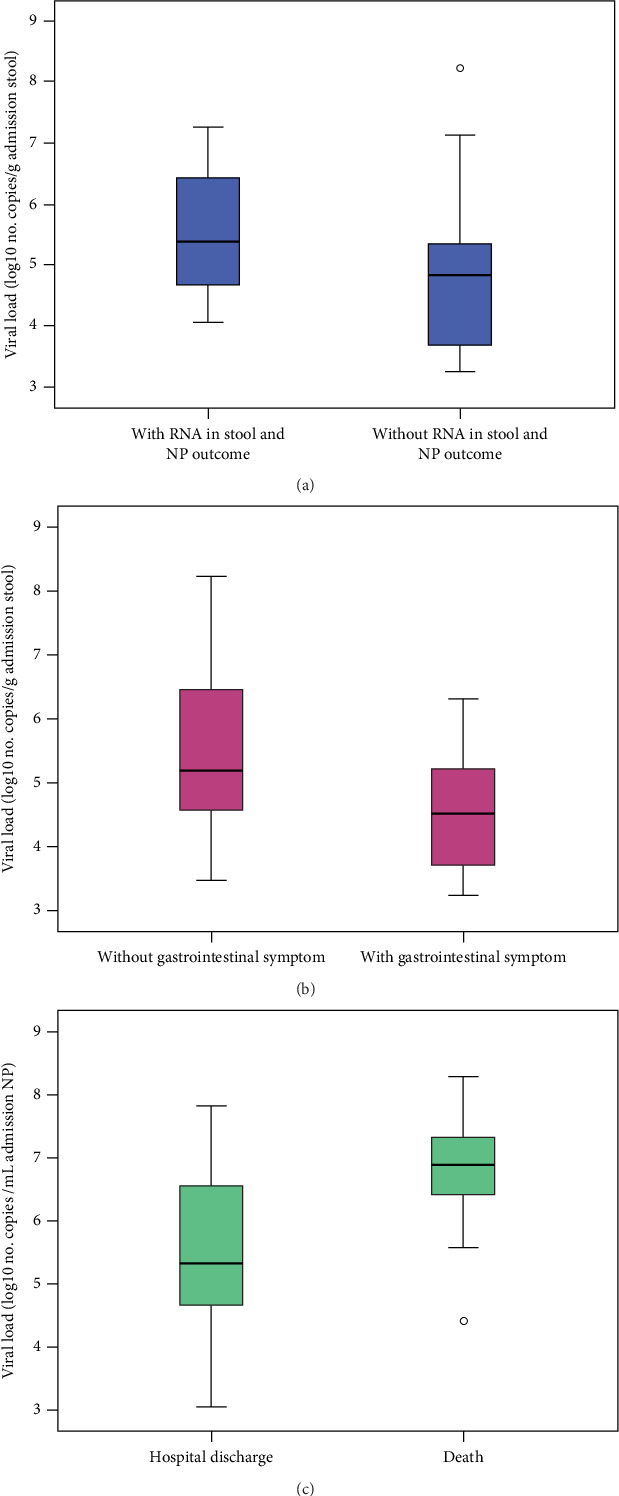
Viral load in the stool or nasopharyngeal/oropharyngeal samples at admission in patients in different situations. The viral load in the stool at admission in patients with and without detectable RNA in the outcome stool and nasopharyngeal/oropharyngeal samples (NP) (*p* = 0.045) (a) and with or without gastrointestinal symptoms (*p* = 0.049) (b). The NP viral load at ICU admission in patients who were discharged or died (*p* = 0.004) (c). Viral load measurements are presented on a log_10_ scale to reduce distortion from extreme values and enhance visualization of proportional variations. Outliers are represented to capture the full range of data reflecting individual-specific responses.

Patients who tested positive for both stool and NP RNA at outcome had a significantly higher stool viral load at admission (*p* = 0.045), whereas NP viral load at admission showed no significant difference (*p* > 0.05) (Figure 4a). The quantified viral loads from the stool and NP samples collected upon admission and at outcome were compared with all the qualitative variables in Table 1 and Table S1. The NP viral load at admission was higher in patients > 65 years (*p* = 0.031), those without odynophagia (*p* = 0.042), those with diarrhea (*p* = 0.047), those requiring an O_2_ mask during hospitalization (*p* = 0.041), and those who died (*p* = 0.004) (Figure 4c). Similarly, at outcome, NP viral load was significantly higher in patients > 65 years (*p* = 0.029), those with > 2 comorbidities (*p* = 0.017), those with chronic kidney disease (*p* = 0.033), those presenting general condition decline as a symptom (*p* = 0.028), and those with creatinine levels > 1.3 mg/dL (*p* = 0.013) or CRP > 100 mg/mL (*p* = 0.005) at admission.

**Figure 5:**
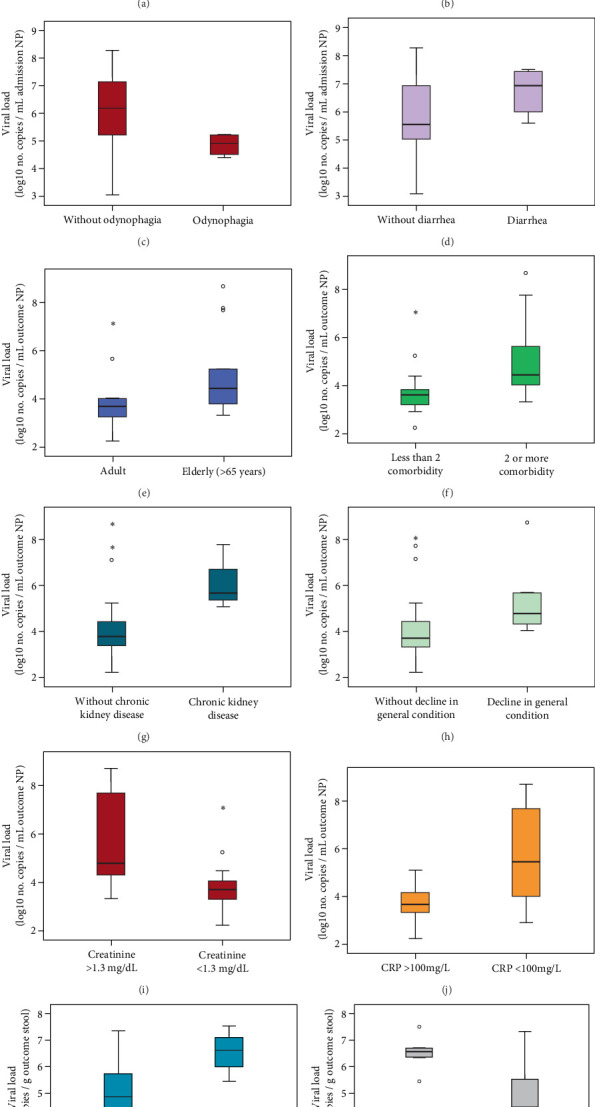
Viral loads from the stool and NP samples collected upon admission and at outcome compared with the qualitative variables. The NP viral load upon admission was compared with elderly patients aged older than 65 years (*p* = 0.031) (a), those utilizing an O_2_ mask during hospitalization (*p* = 0.041) (b), those without odynophagia (*p* = 0.042), (c) and those with diarrhea (*p* = 0.047) (d). The NP viral load outcome was significantly greater in patients older than 65 years (*p* = 0.029) (e) with more than two comorbidities (*p* = 0.017) (f), in those with chronic kidney disease (*p* = 0.033) (g), in those showing a decrease in general condition as a symptom (*p* = 0.028) (h), and in those whose creatinine levels exceeded 1.3 mg/dL (*p* = 0.013) (i) and CRP surpassed 100 mg/mL (*p* = 0.005) (j) at admission. The viral load in the stool at the outcome was greater in patients who exhibited a decrease in their general condition as a symptom (*p* = 0.034) (k) and had an admission laboratory test indicating that their creatinine level was higher than 1.3 mg/dL (*p* = 0.007) (l). NP, nasopharyngeal/oropharyngeal swab; g, grams; CRP, C-reactive protein. Viral load measurements are presented on a log_10_ scale to reduce distortion from extreme values and enhance visualization of proportional variations. Outliers are represented to capture the full range of data reflecting individual-specific responses.

At admission, stool viral load was lower in patients with GI symptoms (*p* = 0.049) (Figure 4b). At outcome, it was higher in patients with general condition decline (*p* = 0.034) and those with creatinine > 1.3 mg/dL at admission (*p* = 0.007). Box plots comparing viral loads from NP and stool samples at admission and outcome across significant clinical variables are shown in an additional file (Figure 5).

In 18 patients, additional stool samples were collected during hospitalization; three were persistently negative (one deceased, two discharged). In four patients, stool RNA was detected only in the first sample but became undetectable or unquantifiable in subsequent collections and these cases were excluded from further analysis.

**Figure 6:**
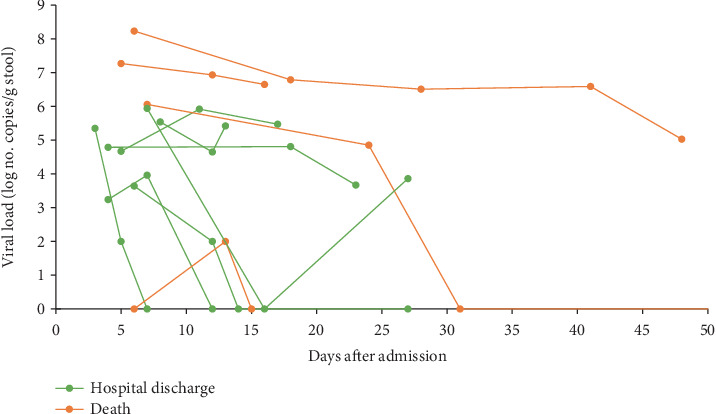
Base-10 logarithmic viral load of stool during patients' hospitalization and outcomes. Viral load measurements are presented on a log_10_ scale to reduce distortion from extreme values and enhance visualization of proportional variations.

Figure 6 illustrates stool viral load trajectories for 11 patients with intermediate quantifiable samples. One patient had an initial negative sample that later tested positive. In two patients discharged from the hospital, viral load decreased and subsequently increased, while one case became undetectable. No consistent trend in stool viral load changes was observed among discharged or deceased patients. Overall, NP viral load at admission was two base-10 logarithms higher than that in stool samples, based on total eluted volume (*p* = 0.005).

## 4. Discussion

Viral load and shedding dynamics are influenced by both viral and host factors, including viral variants, patient age, sex, and immune status [16]. The increase in population immunity through natural infection and vaccination has driven rapid viral evolution, leading to the emergence of new variants that, among other characteristics, exhibit changes in viral load compared to the ancestral strain. Understanding viral load has important implications for viral pathogenesis and public health policies, particularly regarding population screening, antiviral treatment, and isolation periods [13, 16].

In this study, we investigated viral loads in respiratory and stool samples from critically ill ICU patients, tracking their clinical progression and outcomes in a public hospital in southeastern Brazil—a reference center for SARS-CoV-2 treatment. We found that nearly 40% of patients had RNA detection in both stool and nasopharyngeal samples at the time of clinical outcome. A higher NP viral load at admission was associated with unfavorable outcomes, while stool viral load at admission was greater among patients without GI symptoms. Additionally, patients with diarrhea exhibited a more rapid decline in NP viral load.

The patients included in this study progressed to severe disease (ICU admission) within 10 days of symptom onset, with most being male and having comorbidities, consistent with previous reports [17–19]. A recent international study identified age as the most significant determinant of mortality risk, with pre-existing comorbidities and male sex also associated with increased mortality [19]. The most commonly reported symptoms in our cohort—cough, shortness of breath, and fever—align with those frequently described in COVID-19, reinforcing the respiratory tract as the primary system affected [17, 20, 21]. The need for ventilation upon admission and during hospitalization was widespread among our study population, which aligns with severe cases characterized by an obliterative pattern of lung injury, increasing the demand for respiratory support [22].

In this study, RNA detection in stool samples at admission was notably high (89%), contrasting with the pooled prevalence of 46.8% reported in a systematic review of 46 studies by Zhou et al. [23]. Among the 21 studies in that review that included more than 30 COVID-19 patients, only three reported stool RNA detection rates exceeding 65%. These differences may be attributed to patient profiles, as severe cases have been shown to exhibit higher stool viral RNA detection rates than milder cases [24, 25]. In contrast, the prevalence of GI symptoms in our cohort was similar to that described by Zhou et al. and other studies [8, 26], although higher frequencies of GI symptoms have also been reported [9].

Viral RNA detection in stool was associated with a hospital stay of less than 15 days, suggesting that ICU length of stay may serve as an additional host response factor. One of the most severe consequences of COVID-19 is the excessive activation of the immune system, leading to a cytokine storm—an intense inflammatory response that can cause severe systemic damage and multiple organ failure [27]. The absence of RNA in stool at the time of clinical outcome was correlated with prolonged hospitalization and the need for invasive ventilation. These factors may contribute to greater patient decompensation due to secondary infections, ultimately leading to unfavorable outcomes. In our study, 75% of the patients who died had been hospitalized for more than 15 days (data not shown).

The median stool viral load found in this study fell within the range of the medians described in other studies [8, 14], whereas the NP viral load was 10 times higher than previously reported [8]. Differences in patient clinical and sociodemographic profiles, as well as variations in study design, may have contributed to these discrepancies. Additionally, the lack of methodological standardization across studies hinders direct comparisons. Viral clearance in the digestive system occurs more slowly than in the respiratory tract, raising concerns about the potential fecal infectivity of SARS-CoV-2 in the later stages of infection and the persistence of viral RNA in recovered COVID-19 patients [9, 23, 28]. At clinical outcome, more patients remained NP-positive than stool-positive. However, while NP viral load declined over the course of illness, stool viral load increased. This pattern may indicate slower clearance in the intestinal tract, a delayed onset of intestinal infection, or persistent viral infectivity, as previously reported [23, 28].

Notably, we found that NP viral load declined faster in patients with diarrhea, suggesting a possible relationship between GI immune activation and respiratory infection control. SARS-CoV-2 infection in human intestinal cells triggers a robust innate immune response mediated by type III interferon, inhibiting viral replication [7]. This response also extends to regulating infections in other organ systems. The production of a diverse array of cytokines within human intestinal organoids may contribute to overall systemic cytokine levels, signifying a multifaceted inflammatory response that may impact patient prognosis [6]. Although our results contribute novel insights by demonstrating an association between diarrhea and accelerated nasopharyngeal viral clearance, this observation does not establish a causal relationship. Other factors, such as differences in immune activation, the gut-lung axis, or variations in treatment regimens (e.g., antivirals or immunomodulators), could also play a role. Further research is necessary to disentangle the mechanisms underlying these associations and their potential impact on clinical outcomes.

Our results highlighted that NP RNA viral load at admission was significantly higher in older patients (aged > 65 years), those requiring oxygen support during hospitalization, and those who died. When assessing the difference in viral load measured from nasopharyngeal swabs at outcome, higher viral loads were associated with renal disease or renal dysfunction at admission (creatinine > 1.3 mg/dL). This finding suggests that host response dysregulation influences respiratory infection control. Although chronic diseases are critical risk factors, SARS-CoV-2 can also directly affect the kidneys by binding to ACE2 receptors and causing cellular damage or indirectly through cytokine storms and nephrotoxic drug use [27, 29]. Cytokine storm–related renal damage may, in turn, influence stool viral loads, as patients with elevated creatinine levels exhibited higher stool RNA loads at outcome.

We found that the viral load at admission was lower in the stools of patients with GI symptoms, and the presence of RNA in the stools at admission or outcome showed no relationship with these symptoms. In fact, the association between GI symptoms or diarrhea and SARS-CoV-2 RNA detection is controversial [8, 9, 26, 30]. Early GI tract disturbances may result from gut microbiota imbalances, which play a key role in COVID-19, rather than direct epithelial cell damage [27, 31]. A higher NP viral load at admission correlated with diarrhea symptoms, suggesting that GI infection may depend on pre-existing respiratory infection. Studies have demonstrated the presence of detectable SARS-CoV-2 RNA or nucleocapsid protein in the GI tracts of intranasal inoculated animals [6, 32, 33]. Although it is not entirely clear how SARS-CoV-2 reaches the intestine, it may occur through the bloodstream or lymphatic system or via an intragastric route protected by mucus or GI contents [33]. Jiao et al. [33] demonstrated that intranasal inoculation of rhesus macaques resulted in a greater viral load in both respiratory and digestive tissues compared to intragastric inoculation.

Our study indicated that viral loads peaked at infection onset and decreased over time, possibly due to differences in intestinal infection dynamics and host response. Studies analyzing multiple stool samples from the same patients have reported fluctuations in viral load throughout SARS-CoV-2 infection, including undetectable and redetectable RNA [34–36]. Interestingly, our data showed that only patients with favorable outcomes exhibited an unexpected decrease followed by an increase in viral load, warranting further investigation into viral pathogenesis and elimination dynamics across different clinical outcomes.

This study holds several limitations, including those related to representativeness, partly due to challenges in obtaining stool samples near admission and outcome from bedridden patients with limited oral intake and irregular bowel movements. This led to the exclusion of a significant number of patients and may have introduced selection bias and reduced the representativeness of our cohort. Consequently, the power of multivariate analysis to fully explore the association between viral load dynamics and clinical outcomes is limited. Additionally, molecular biology techniques used for stool specimen analysis may yield false negatives, and statistical analyses did not account for diverse medications used in ICU patients. Despite these limitations, our findings provide valuable insights and represent data from a prospective study conducted in a reference center for COVID-19 care in southeastern Brazil, with sample collection and analysis from two distinct sites (NP swabs and stool). These results may contribute to future research and discussions on this topic.

## 5. Conclusions

Our findings reveal associations between viral load and clinical conditions in patients with severe COVID-19. Notably, diarrhea correlated with a faster decline in viral load in nasopharyngeal samples. Additionally, viral clearance in stool samples was more pronounced than in respiratory samples. These results underscore the importance of monitoring different sample types to capture the complexities of viral behavior and patient response in COVID-19.

## Figures and Tables

**Table 1:** Hospitalization characteristics of ICU patients grouped by stool RNA presence/absence at outcome.

	**Patients ** **n** = 46	**RNA in stool at the outcome**		**p** ^**b**^
		**Positive ** **n** = 21	**Negative ** **n** = 25	
Hospitalization period, median (days)	16 (2–96)	13 (2–48)	17 (4–96)	**0.022**
Infirmary hospitalization period, median (days)	2 (1–18)	2 (1–18)	3 (1–17)	0.757
ICU hospitalization period, median (days)	16 (2–96)	12 (2–30)	17 (3–96)	**0.010**
Hospitalization period of more than 15 days	25 (54.3%)	8 (38.1%)	17 (68.0%)	**0.043**
Ventilation during hospitalization	45 (97.8%)	20 (95.2%)	25 (100%)	0.538
Catheter	27 (58.7%)	14 (66.7%)	13 (52.0%)	0.377
Duration of usage, median (days)	4 (1–14)	4 (2–9)	2 (1–14)	0.325
O_2_ mask	25 (54.3%)	11 (52.4%)	14 (56.0%)	1.000
Duration of usage, median (days)	3 (1–12)	1 (0–10)	2.5 (1–12)	0.635
Noninvasive ventilation	27 (58.7%)	13 (61.9%)	14 (56.0%)	1.000
Duration of usage, median (days)	3 (1–12)	6 (1–12)	3 (1–10)	0.085
Orotracheal intubation	26 (56.5%)	8 (38.1%)	18 (72.0%)	**0.021**
Duration of usage, median (days)	16 (4–24)	12 (8-24)	16 (4–23)	0.253
Tracheostomy	12 (26.1%)	1 (4.8%)	11 (44.0%)	**0.003**
Duration of usage, median (days)	12.5 (1–73)	10 (10)	13 (1–73)	0.663
Outcome				0.538
Hospital discharge^a^	30 (65.2%)	15 (71.4%)	15 (60.0%)	
Death	16 (34.8%)	6 (45.7%)	10 (40.0%)	

*Note:* Bold indicates statistical significance (two-sided *p* < 0.05).

^a^Patients who were transferred from the hospital to long-stay facilities were considered discharged (*n* = 1).

^b^Statistical analyses were performed using Pearson's chi-square or Fisher's exact tests, independent continuous variables were conducted using the Mann–Whitney tests, and *p* < 0.05 was considered significant.

## Data Availability

The data that support the findings of this study are available from the corresponding author upon reasonable request.

## Supporting Information

Supporting Information Additional supporting information can be found online in the Supporting Information section. Table S1. Sociodemographic and clinical characteristics at ICU admission grouped by stool RNA presence/absence at outcome. Table S2. SARS-CoV-2 RNA in stool specimens and naso/oropharyngeal swabs at outcome of 46 ICU patients.
