# Supplementary material for: SARS-CoV-2 Viral Load in Stool and Nasopharyngeal/Oropharyngeal Samples: Implications for Clinical Progression in Severe COVID-19 Patients
Source: Int J Microbiol. 2025 Oct 10;2025:1501327. doi: 10.1155/ijm/1501327 (PMC12534152; doi:10.1155/ijm/1501327)
Supplement: Supporting Information — Additional supporting information can be found online in the Supporting Information section. Table S1. Sociodemographic and clinical characteristics at ICU admission grouped by stool RNA presence/absence at outcome. Table S2. SARS-CoV-2 RNA in stool specimens and naso/oropharyngeal swabs at outcome of 46 ICU patients. [file 1501327.f1.pdf]

Supplementary Material

Table S1 –Sociodemographic and clinical characteristics at ICU admission grouped by stool RNA presence/absence at outcome.

	Patients n=46	RNA in stool at the outcome		p^d
		Positive n=21	Negative n=25	
**Age, years (median)**	61 (30–88)	61 (31–88)	61 (30–87)	0.716
Older adults (>60 years)	26 (56.5%)	11 (52.4%)	15 (60.0%)	0.766
Older adults (>65 years)	19 (41.3%)	9 (42.9%)	10 (40.0%)	1.000
**Sex**				0.149
Male	25(54.3%)	14 (66.7%)	11 (44.0%)	
Female	21 (45.7%)	7 (33.3%)	14 (56.0%)	
**Ethnicity**				0.620
White	16 (34.8%)	8 (38.1%)	8 (32.0%)	
Black	7 (15.2%)	2 (9.5%)	5 (20.0%)	
Multiracial	23 (50.0%)	11 (52.4%)	12 (48.0%)	
**Comorbidities**	40 (87.0%)	18 (85.7%)	22 (88.0%)	1.000
2 or more comorbidities	23 (50.0%)	10 (47.6%)	13 (52.0%)	1.000
Hypertension	28 (60.9%)	10 (47.6%)	18 (71.0%)	0.132
Diabetes	15 (32.6%)	7 (33.3%)	8 (32.0%)	1.000
BMI>30	17 (37.0%)	6 (28.6%)	11 (44.0%)	0.363
BMI median (range)	27.3 (18–46)	27.34 (18–45.7)	27.22 (21.5–43.3)	0.774
Chronic kidney disease	3 (6.5%)	2 (9.5%)	1 (4.0%)	0.585
Chronic respiratory disease	1 (2.2%)	1 (4.8%)	0 (0.0%)	0.457
Cardiovascular disease	8 (17.4%)	4 (19.0%)	4 (16.0%)	1.000
Other[a]	2 (4.5%)	0 (0.0%)	2 (8.0%)	0.493
**Signs and symptoms**				
Generalized weakness	7 (15.2%)	4 (19.0%)	3 (12.0%)	0.686
Dyspnea	44 (95.7%)	19 (90.5%)	25 (100.0%)	0.203
Desaturation	40 (87.0%)	16 (76.2%)	24 (96.0%)	0.079
Fever	29 (63.0%)	14 (66.7%)	15 (60.0%)	0.762
Cough	29 (63.0%)	13 (61.9%)	16 (64.0%)	1.000
Odynophagia	4 (8.7%)	1 (4.8%)	3 (12.0%)	0.614
Myalgia	16 (34.8%)	8 (38.1%)	8 (32.0%)	0.760
Adynamia	9 (19.6%)	4 (19.0%)	5 (20.0%)	1.000
Anosmia/Dysgeusia	8 (17.4%)	2 (9.5%)	6 (24.0%)	0.260
Headache	6 (13.0%)	4 (19.0%)	2 (8.0%)	0.390
Diarrhea	8 (17.4%)	4 (19.0%)	4 (16.0%)	1.000
Vomiting	3 (6.5%)	1 (4.8%)	2 (8.0%)	1.000
Nausea	3 (6.5%)	1 (4.8%)	2 (8.0%)	1.000
Gastrointestinal symptom[b]	12 (26.1%)	5 (23.8%)	7 (28.0%)	1.000
Total 4 or more symptoms	36 (78.6%)	16 (76.2%)	20 (80.0%)	0.516
SpO_2 <90 admission ICU	25 (54.3%)	10 (50.0%)	15 (60.0%)	0.557
**Oxygen support on admission**				0.860
None (room air)	1 (2.2%)	1 (4.8%)	0 (0.0%)	
Noninvasive ventilation	32 (69.6%)	14 (66.7%)	18 (72.0%)	
Invasive ventilation	13 (28.3%)	6 (28.6%)	7 (28.0%)	
**Laboratory findings**				
Leucocytes (>15000)	8 (17.4%)	4 (19.0%)	4 (16.0%)	1.000
Platelets (<100.000)	3 (6.6%)	2 (9.5%)	1 (4.0%)	0.585
Creatinine (>1.3 mg/dL)	14 (30.4%)	6 (28.6%)	7 (28.0%)	1.000

C-Reactive Protein, CRP (>100 mg/L)	32 (69.6%)	13 (61.9%)	19 (76.0%)	0.349
D-dimer (>1.5 mg/L)[c]	18(39.1%)	6 (28.6%)	12 (48.0%)	0.181

[a]One with cancer and one with autoimmune disease. [b]At least one of the symptoms: diarrhea, vomiting, or nausea. [c]No available data for 10 patients. [d]Statistical analyses were performed using Pearson's chi-square or Fisher's exact tests, independent continuous variables were conducted using the Mann-Whitney tests, $p < 0.05$ were considered significant.

Table S2 – SARS-CoV-2 RNA in stool specimens and naso/oropharyngeal swabs at outcome of 46 ICU patients.

Viral RNA at the outcome		Stool		Total	*p*
		Detectable N (%)	Negative N (%)		
Nasopharyngeal/ oropharyngeal	Detectable	18 (39.1)	13 (28.3)	31 (67.4)	
	Negative	3 (6.5)	12 (26.1)	15 (32.6)	0.026[a]
	Total	21 (45.7)	25 (54.3)	46 (100)	

[a]Fisher's exact test
